# Monthly Summary

**Published:** 1874-01

**Authors:** 


### MONTHLY SUMMARY.

---

*Listening with the Teeth.*—Listening with your teeth may seem a comical action, but it is a possible one, and after all, no more unnatural than that of talking with the fingers, which every educated mute can perform. Any one who will hold a vibrating tuning-fork to his dentals will be struck with the sonorous thrill that goes through his head, and the superior intensity of the sound compared to what is experienced when the fork is held to the ear.

That other nerves than those appointed for audition are capable of conveying sound vibrations to the brain, is thus pretty evident. We had remarked this so often that we were fully prepared to give credence to a statement made by a newspaper correspondent, to the effect that a deaf friend was stirred with delight at the music of a violin, rendered audible to him first by placing the instrument between his teeth while it was played upon, and afterwards by means of a string tied to the violin, one end of which was held in the deaf man's mouth. The writer in question suggests a repetition of his experiment upon a larger scale, with a number of strings stretched from an orchestral sounding-board to the mouths of a deaf audience. A concert of such character might be a ludicrous affair to sharp-eared spectators, but we venture to think with the proposer, that the experiment would be far more gratifying than absurd to those who, for the first time in their lives, were thus moved by concord of sweet sounds. Let us hope that curiosity, if nothing else will prompt a fair trial by those who have charge of the deaf in our asylums.

As our inventive times go, we ought not to be backward in attacking any problem for the alleviation of bodily suffering or the restoration of natural deformations. We make the lame walk, and the toothless bite. We have even made the blind to see; for lately a Venetian surgeon succeeded in restoring the lost vision of a man whose case had been abandoned as incurable. —*Ex. Druggists Circular.*

---

*The Prevention of Loss of Blood.*—The *Medical Times and Gazette* recommends the following plan to diminish the loss of blood in operations:—

An elastic bandage, about two inches and a half in width and from five to ten yards long, is firmly bound round the limb, commencing at the toes or fingers, as the case may be, and is then continued upwards so as to drive the blood before it out of the veins and arteries. When the desired point has been reached, a strong india-rubber band, about half an inch in diameter, is tightly drawn two or three times round the limb, just above the elastic bandage, and fastened by hooks. The bandage is then removed, leaving the tissues blanched and ensanguined. Not a particle of blood is lost during the operation, which is really more bloodless than when performed on the dead subject. After the operation is completed the rubber rope is removed, and the blood then finds its way into the vessels, which are ligatured or twisted according to the taste or inclination of the surgeon. On this plan, which has been carried out at St. Thomas', Guy's, London, and St. Bartholomew's Hospitals, many operations have now been performed, including excision of the knee and elbow joints, amputations, and the removal of dead bone; and Mr. Wagstaffs has recently amputated through the thigh for gangrene of the foot on this plan, the precaution having been taken to commence the application of the elastic bandage several inches above the mortified part. No ill effects of any kind have hitherto been observed from the use of this contrivance. Although the duration of the operations has varied from a few minutes up to half an hour, and even more, during the whole of which time the circulation has been completely arrested, no evidence has been afforded of the formation of emboli or thrombi in any of the cases. But it is one of the possible evils of the device that the prolonged pressure on the vessels and complete stoppage of circulation may, under certain conditions, lead to the formation of a clot, which, on the re-establishment of circulation, may be carried along the vessels, and arrested in some part of their course, giving rise to circumscribed inflammation or even gangrene. There is also considerable danger in applying the bandage over parts which are inflamed and suppurating, especially if decomposition be going on, lest some of the clots which are found in the blood-vessels of the affected parts be detached and forced into the blood current. For such cases it would be well to employ in addition a modification of the plan which has been practiced at Edinburgh for the last two or three years, and which consists in suspending the limb for some minutes before the operation, so that the blood may gravitate downwards. Then the bandage may be applied at the proximal side of the diseased part, thus avoiding all risks of septic poisoning or of embolism.

---

*Mercury in the System.*—Prof. Hyatt delivered a lecture on mercury, in Vienna recently, when he exhibited the leg-bone of a man whose death had undoubtedly been hastened by mercury. On striking the bone heavily upon the table, out fell thousands of little glittering globules of mercury—bright metalic mercury —which rolled about upon the black surface before him, collecting here and there into drops. This mercury had been absorbed during life, undermined the man's system, and proved fatal to him. The mortality among those who work in mines of quicksilver, or in the works where it is reduced, is known to be frightful. In the celebrated mines of Idria, the men work alternately one month in the mines and one month in the smelting-house. But notwithstanding this, it appears that of the hundreds employed there, one-fourth become salivated.—*Am. Chemist.*

---

*Lead Poisoning through Drinking Water.*—M. Leblanc, of Paris, has been examining water conveyed through leaden pipes. He tested five litres of such water condensed by evaporation, and the most sensitive re-agents failed to detect it in the presence of any salts of lead. He states that lead is readily affected by distilled water, the result being carbonate of lead, which renders the water milky; but the presence of an infinitely small quantity of carbonate of lime is sufficient to remove from the water its action on the lead. Even rain-water, which absorbs lime from the atmosphere, or that which it meets with on the roofs of houses, contains a sufficient quantity of that substance to render lead unattackable. This would explain why leaden pipes last almost indefinitely without being deteriorated, whereas iron and other metalic pipes are rendered completely useless in the course of a few years, from being corroded and pierced through.—*Med. and Surg. Reporter.*

---

*Hope for the Bald.*—A writer to an English exchange gives the following case, which will be read with interest by that large class of gentlemen whose hair is thin, but not from years :—

A gentleman, who had lost nearly all his hair after a very severe attack of fever, consulted a French physician of great reputed success as a hair restorer. The prescription was a drachm of the homœopathic tincture of phosphorus to one ounce of castor oil; the bare spot to be rubbed with this mixture three times weekly for half an hour each time, after the skin of the head had been thoroughly cleansed with warm water without soap. This treatment was faithfully carried out for about six months; the hair soon began to grow, and in a year from the time of first following the doctor's advice, his head was as thoroughly covered as ever, the new crop of hair being about two shades darker than the old.—*Med. and Surg. Reporter.*

---

*Modification of the Operation for Harelip.*—On two successive Saturdays Sir William Fergusson has recently demonstrated to the student of King's College, London, a model modification of the ordinary operation for hare-lip. The cases in which he carried out this plan were of the usual type, the fissure being as in the majority of instances, on the left side; and in both it was considered advisable to take away the intermaxillary bone. This adds to the success of the operation, not only by removing an occasional obstacle to primary union, but because the teeth, which are subsequently developed from the protecting knob, are worse than useless, by reason of their deficient development and faulty position. Instead, however, of removing the portion of bone readily with the knife or bone forceps, it had occurred to Sir William that it would be much better to operate subcutaneosly, so to speak, by stripping off and retaining the mucous membrane; and accordingly this was done, with perfect success, the bone shelling out readily from its investment. Not only will this procedure greatly accelerate the subsequent process of healing, but the advantage is obvious, of retaining a thick and firm mucous surface in preference to the more artificial substitute of cicatricial tissue.—*Brit. Med. Journal.*

---

*Bad Tea.*—The question of spurious or injurious tea seems to be growing important. The Chinese have discovered that the tea-leaves mixed with dung, iron filings and other substances, all powdered fine, suits the English market, and are sending compounds of that sort over in huge masses, of course not without connivance from some English dealers on this side. On Tuesday, it was alleged by the Sanitary Commissioners of the city (London) that no less than 10,000,000 pounds of such tea, totally unfit for human food, was in bond ready for sale. The quantity would, we believe, be greatly increased if partially adulterated tea were added to the list.—*Spectator.*

*Facial Paralysis.*—An interesting illustration of this common malady was noticed in a man above 45 years of age. No cause could be found for the occurrence of the lesion. The man awoke in the morning and found his face distorted. Upon close examination it was found that there was loss of smell upon the same side of the nose with the paralysis, and also loss of taste upon one half of the tongue upon the same side, thus enabling his medical attendants to arrive at quite definite conclusions with regard to the seat of the lesion.

There was some tenderness over the mastoid region, along the course of the nerve. Iodide of potassium, leeches, and electricity form the main features of treatment. In some of these cases the constant current will act, and the primary current will not; but in this case the constant current would not act, but the primary current would.—*Bellevue Hospital Report in Medical Record.*

---

*On the Surgical Use of Gastric Juice in the Treatment of Cancerous Tumors.*—Professor Lussana, after several years of experiments and observations, has arrived at the following conclusion: 1. Gastric juice applied to these tumors softens their tissues, but its action is not powerful enough to arrest the progress of the disease. 2. That natural gastric juice is more reliable than the artificial. 3. It does not destroy the vascular tunics, but arrests local hemorrhage produced by the destruction of tissues. 4. The gastric juice of dogs was found to act slowly; indeed, it did not much retard the development of the tumor. 5. There is a continuous exhaustive loss of substance during treatment, and it is probable that through the cancerous elements are dissolved by this remedy, the further extension of the disease is not prevented.—*Gion. Ven. di Sci. Med.*

---

*Test for Impurities in Water.*—An English technical periodical says, "Good water should be free from color, unpleasant odor and taste, and should quickly afford a lather with a small portion of soap. If half a pint of water be placed in a perfectly clean, colorless glass-stoppered bottle, a few grains of the best lump white sugar added, and the bottle freely exposed to the daylight in the window of a warm room, the liquid should not become turbid, even after exposure for a week or ten days. If the water become turbid, it is open to the grave suspicion of sewerage-contamination; but if it remains clear, it is almost certainly safe. We owe to Heisch this simple valuable, but hitherto strangely neglected test."—*Medical Times.*

